# Long term surgical outcomes of unilateral recession-resection versus bilateral lateral rectus recession in basic-type intermittent exotropia in children

**DOI:** 10.1038/s41598-021-98801-3

**Published:** 2021-09-29

**Authors:** Dong Hyun Kim, Hee Kyung Yang, Jeong-Min Hwang

**Affiliations:** Department of Ophthalmology, Seoul National University College of Medicine, Seoul National University Bundang Hospital, 82, Gumiro 173 Beon-gil, Bundang-gu, Seongnam-si, Gyeonggi-do 13620 South Korea

**Keywords:** Ocular motility disorders, Eye abnormalities

## Abstract

The long-term results of surgical treatment of intermittent exotropia (X(T)) according to the type of surgery are controversial. We conducted a retrospective cohort study to compare the long-term results between unilateral recession-resection (RR) and bilateral lateral rectus recession (BLR) with an average follow-up of 9.5 years in children with basic-type X(T). Patients with basic-type X(T), who underwent RR (RR group) or BLR (BLR group) and were followed-up for more than 5 years postoperatively, were analyzed. Of the 560 patients, 363 patients received BLR and 197 patients underwent RR. There was no significant difference in the success rates between the two groups until postoperative 3 years. At an average of 9.5 ± 2.6 years after surgery, the success rate of the RR group was significantly higher than that of the BLR group starting from the fourth post-operative year until the last follow-up examination (64.5% vs 43.3%, P < 0.001). By multivariate analysis, preoperative hyperopia of more than + 2.00 diopters, younger age of onset, younger age at surgery, larger exodeviation at near than at distance of > 5 prism diopters, and the type of surgery (BLR) were risk factors of recurrence. In conclusion, RR was more successful than BLR with a lower recurrence rate in the long-term follow-up of patients with basic-type X(T).

## Introduction

Intermittent exotropia (X(T)) is the most common form of divergent strabismus^1^, and the prevalence of exotropia is much higher in Asians than in other ethnic groups^2–4^. Although X(T) is the most common exotropia, it remains to be fully understood. In particular, the success rate for surgical intervention in X(T) is highly variable, ranging from 33 to 83%^5–10^. Furthermore, a surgical method yielding optimal long-term outcome remains controversial^5–16^.

There have been many studies on the surgical results of unilateral lateral rectus recession with medial rectus resection (RR) and bilateral lateral rectus recession (BLR)^8,17–24^. In three randomized control studies^5,11,12^, the success rate of RR was better than that of BLR at the 1 year follow–up point. A recent pediatric eye disease investigator group (PEDIG) study reported no difference in the success rates between RR and BLR within the first three years after surgery^13^. A meta-analysis of studies performed up to June 2017 showed that RR was more successful in basic-type X(T)^14^. Conversely, there are several retrospective studies in which BLR has a better success rate than RR^6,9,15,16^.

The cumulative rate of recurrence after surgical intervention in X(T) gradually increases with time^25^. However, most of the previous studies had a short observation period^5,11,12^; and those with a long follow-up period were limited with having a small number of included patients^26–28^. Herein, we analyzed the long-term postoperative results between RR and BLR with an average follow-up period of 9.5 years in a large scale of children between the age of 3 and 15 years with basic-type X(T).

## Results

### Patient demographics

Among the 560 patients with basic-type X(T), 363 patients received BLR (BLR group) and 197 patients underwent RR (RR group). The preoperative patient characteristics were not significantly different between the two groups (Table 1), except for fixation preference, which was more common in the RR group compared to the BLR group (52.8% vs 19.3%, P < 0.001).

**Table 1:** Patients’ characteristics in the bilateral lateral rectus recession (BLR) group and unilateral lateral rectus recession–medial rectus resection (RR) group.

Characteristics, mean ± SD (range)	BLR group (n = 363)	RR group (n = 197)	P value
Gender (M:F)	164:199	87:110	0.817^a^
Age at onset (y)	3.7 ± 2.5 (0–12)	4.0 ± 2.6 (0–10)	0.178^b^
Age at surgery (y)	6.3 ± 2.5 (3–15)	6.2 ± 2.4 (3–15)	0.631^b^
Follow-up period (y)	9.5 ± 2.6 (5.0–15.0)	9.6 ± 2.5 (5.0–15.5)	0.547^b^
Best corrected visual acuity (LogMAR)	0.18 ± 0.17 (0–0.82)	0.17 ± 0.15 (0–0.82)	0.598^b^
Spherical equivalent refractive errors (D)	− 0.19 ± 1.78 (− 7.75 to + 6.50)	− 0.18 ± 1.95 (− 8.00 to + 8.50)	0.937^b^
Stereopsis (LogArcsec)	1.94 ± 0.17 (1.60–2.60)	1.98 ± 0.21 (1.60–2.60)	0.064^b^
Distance deviation (PD)	29.2 ± 7.6 (15–68)	29.1 ± 7.5 (16–75)	0.888^b^
Near deviation (PD)	30.0 ± 8.0 (15–70)	29.1 ± 7.9 (8–70)	0.223^b^
Fixation preference, n (%)	70 (19.3%)	104 (52.8%)	< 0.001^a^
Lateral incomitancy, n (%)	53 (15.4%)	30 (16.1%)	0.827^a^
Constancy of deviation at distance, n(%)	144 (39.7%)	95 (48.2%)	0.051^a^
Constancy of deviation at near, n(%)	87 (24.0%)	53 (26.9%)	0.443^a^

BLR: bilateral lateral rectus recession; RR: unilateral lateral rectus recession and medial rectus resection; M: male; F: female; y: years; LogMAR: Logarithm of the minimum angle of resolution; LogArcsec: Log arcsecond; D: diopters; PD: prism diopters.

^a^P value by Chi-square test, ^b^Student t test.

### Surgical outcomes

**Figure 1:**
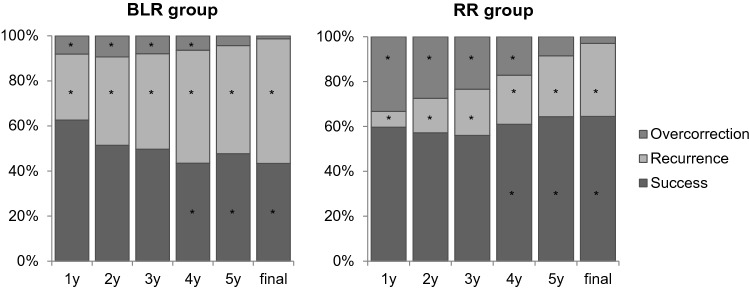
The proportions of surgical outcomes in the bilateral lateral rectus recession (BLR) group and unilateral lateral rectus recession-medial rectus resection (RR) group for basic-type intermittent exotropia. Surgical success rate at each postoperative time up to 3 years after surgery were not different between the groups. However, the success rates were significantly higher in the RR group than in the BLR group from 4 years after surgery (Final success rates 64.5% vs 43.3%, P < 0.001). *P < 0.05 by Chi-square test.

At the final examination after a mean follow-up of 9.5 ± 2.6 years (5.0–15.5), 127 of 198 patients (64.5%) in the RR group had ocular alignment meeting the defined criteria of success, 64 patients (32.5%) had recurrence, and 6 patients (3.0%) had overcorrection (Fig. 1). In the BLR group, 157 of 363 patients (43.3%) had successful alignment, 200 patients (55.2%) had recurrence, and 5 patients (1.4%) were overcorrected.

There was no significant difference in success rates between the two groups until 3 years postoperatively, but the RR group showed higher success rates compared to the BLR group at 4 years (P < 0.001) after operation, and this was maintained until the final examination (P < 0.001). The recurrence rates were lower in the RR group compared to the BLR group at all follow-up periods up to the final examination (P < 0.001). The overcorrection rates were higher in the RR group up to 4 years; however, the rates became comparable in both groups after 5 years (Fig. 1).

**Figure 2:**
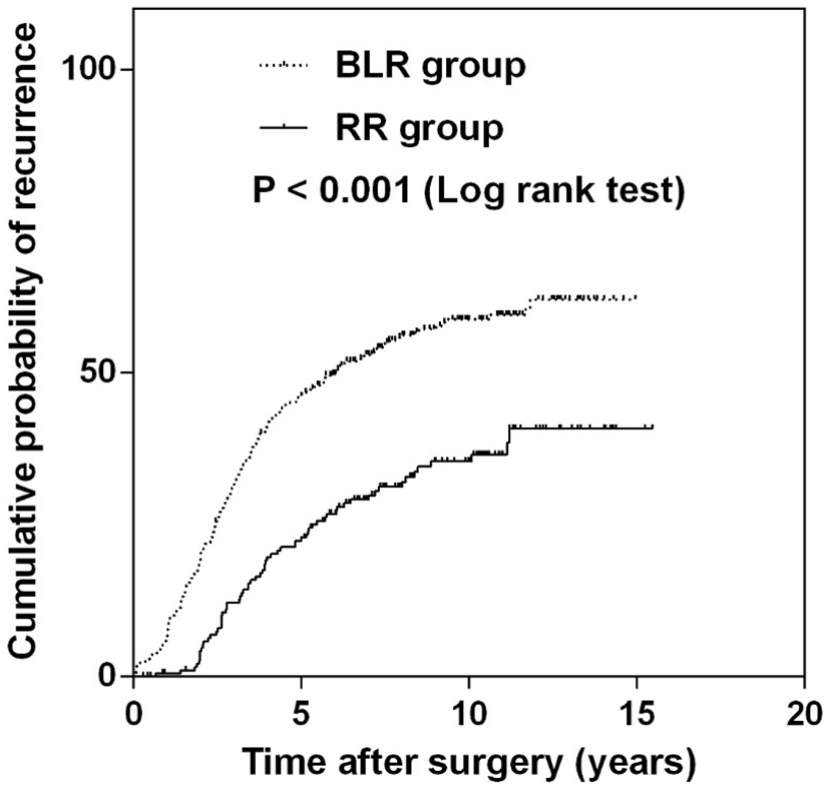
Kaplan–Meier analysis for cumulative recurrence of exodeviation of more than 10 prism diopters (PD) in the bilateral lateral rectus recession (BLR) group and unilateral lateral rectus recession–medial rectus resection (RR) group for basic-type intermittent exotropia. Analysis showed a statistically significant difference in the cumulative probability of recurrence between the groups, with less recurrence in the RR group compared to the BLR group (P < 0.001, log-rank test).

By Kaplan–Meier survival analysis, the cumulative probability of recurrence was lower in the RR group (35.0%) compared to the BLR group (58.3%) at 10 years after surgery (P < 0.001, log rank test) (Fig. 2). The rates of recurrence per person-year were 9.6% in the BLR group and 4.9% in the RR group.

A total of 221 patients (39.5%) eventually required reoperations for recurrence during the follow-up period; 167 patients (46.0%) in the BLR group, 54 patients (27.4%) in the RR group. The reoperation rate was significantly lower in the RR group (P < 0.001). The average number of reoperations was not significantly different between the two groups (1.13 vs 1.11, P = 0.739). The mean interval from the first operation to reoperation was not significantly different between both groups, which were 3.6 ± 2.2 years in the BLR group and 4.1 ± 2.4 years in the RR group (P = 0.122). As for the type of surgery to treat exotropia recurrence, 153 patients underwent unilateral medial rectus muscle resection, and 14 patients underwent bilateral medial rectus muscle resection in the BLR group. In the RR group, 45 patients underwent contralateral RR, and nine patients underwent unilateral lateral muscle recession.

### Risk factors for recurrence

The risk factors associated with recurrence after operation were evaluated. In univariate analysis, male gender, BLR surgery, younger age of onset, younger age at surgery, hyperopia, and larger angle of exodeviation at near than at distance were significant risk factors associated with recurrence. In multivariable analysis, younger age of onset ≤ 4 years of age, younger age at surgery < 6 years of age, preoperative hyperopia > + 2.00 D, larger exodeviation at near than at distance by > 5 PD, and BLR surgery were risk factors for recurrence (Table 2).

**Table 2:** Variables associated with recurrence.

Variable	Univariate analysis		Multivariate analysis	
	OR (95% CI)	P value	OR (95% CI)	P value
Gender (male vs female)	1.6 (1.1–2.2)	0.008		
Age at onset (≤ 4 years vs > 4 years)	2.0 (1.4–2.8)	< 0.001	1.6 (1.0–2.4)	0.046
Age at surgery (< 6 years vs ≥ 6 years)	1.9 (1.3–2.6)	< 0.001	1.6 (1.1–2.5)	0.023
Hyperopia (> + 2.00 D vs ≤ + 2.00 D)	2.3 (1.1–5.0)	0.021	2.5 (1.1–6.0)	0.023
Larger angle at near (> 5 PD vs ≤ 5 PD)	1.9 (1.1–3.1)	0.015	2.1 (1.2–3.5)	0.007
Type of surgery (BLR vs RR)	2.6 (1.8–3.7)	< 0.001	2.7 (1.9–4.0)	< 0.001

OR: odds ratio; CI: confidence interval; D: diopters; PD: prism diopters; BLR: bilateral lateral rectus muscle recession; RR: unilateral lateral rectus recession and medial rectus resection.

## Discussions

Our study evaluated the largest series of patients with basic-type X(T) operated by a single surgeon, with an average follow-up of 9.5 years. In this study, basic type X(T) patients who underwent RR (the RR group) showed better results with lower recurrence rates compared with those who underwent BLR (the BLR group). Patients in the BLR group were more likely to relapse within 1 year compared with those in the RR group (29.3% vs 7.0%, P < 0.001). Although the overcorrection rate was higher in the RR group until 4 years after surgery, it continuously decreased over time thereafter, and there was no significant difference in both groups beyond the 5-year point. Other than the type of surgery, patients with younger age of onset, larger exodeviation at near than at distance, and preoperative hyperopia were related to higher risk of recurrence after surgery.

It is well known that the recurrence rates for surgical intervention in X(T) increases as postoperative follow-up time increases^25^. However, most studies reported results within 2–3 years, and only a few studies analyzed long-term observations in X(T). Zibrandtsen et al.^26^ evaluated the results of 25 patients with 10 years of follow-up, and about 50% of patients had good long-term results. Baker et al.^27^ evaluated a 20-year follow-up of X(T) surgery in 30 patients, and about 2/3 required only a single surgery. Pineles et al.^28^ analyzed 50 patients with X(T) who were followed-up for at least 10 years postoperatively and found that 64% of patients showed an excellent motor outcome, including multiple surgeries. In their study, reoperation for recurrent exotropia was done in 48% of patients, which is comparable to our study (39.6% had surgery for recurrence)^28^. However, these former studies were limited by small number of patients, undistinguished type of exotropia, and lack of comparison between different surgical methods.

To the best of our knowledge, four prospective randomized studies^5,11–13^ have compared the surgical outcomes between BLR and RR procedures in patients with X(T). Kushner^5^ randomized 36 patients with basic-type X(T) into two groups—one receiving RR and the other BLR—and reported that patients who underwent the RR procedure had significantly better surgical outcomes with at least 1 year of postoperative follow-up. Somer et al.^11^ analyzed 47 patients, and Zhang et al.^12^ evaluated 116 patients, and both reported better surgical success rates in the RR group compared with the BLR group after 1 year of follow-up. A recent PEDIG study^13^ reported the results of a multicenter randomized clinical trial in 197 children with 3 years of postoperative follow-up, and did not find a significant difference in the suboptimal surgical outcome by 3 years between X(T) children treated with BLR and those treated with RR. In summary, 3 out of 4 randomized clinical trials support RR in basic-type X(T). In addition, a meta-analysis of papers up to June 2017 indicated that the RR procedure is associated with higher success rates and lower recurrence rates in patients with basic-type X(T). Our study is consistent with the studies mentioned above in that the RR procedure is more effective for basic-type X(T). The strength of our results is based on the large number of patients with a longer follow-up period compared with most of the previously published reports^5,11–13^.

Meanwhile, considering retrospective studies, Maruo et al.^6^ reported that BLR produced better outcomes at a 4-year follow-up in 666 patients (66.7% vs 32.8%), but they included different types of X(T) other than the basic-type. Choi et al.^9^ concluded that the surgical outcome at the 2-year follow-up was not different between the two groups in 128 patients; but the outcome after 3.8 years demonstrated a higher success rate in the BLR group than in the RR group (58.2% vs 27.4%). However, in their study, the preoperative angle of deviation was larger in the BLR group than in the RR group, which may affect the surgical dose and surgical outcome. Xie et al.^29^ compared 330 patients for 1 year and found that there was no statistically significant difference in the success rate between the BLR and RR groups (60.6% vs 57.7%), but reported a greater frequency of overcorrection in the RR group. This is similar to our findings in that there was no difference in the initial success rate between the two groups with a higher initial overcorrection in the RR group. However, they compared the results only after 1 year^29^; thus, overcorrection in the RR group may be reduced by exodrift over a long period of observation as in our study.

Burian and colleagues classified exotropia based on distance/near differences^30^. They recommended BLR in divergence excessive-type X(T), and RR or bilateral medial rectal resection in convergence insufficiency-type X(T)^30^. These recommendations are based on the hypothesis that weakening of divergence affects the distance deviation and strengthening of convergence affects the near deviation. Furthermore, Burian^30^ and Jampolsky^31^ observed that in patients with basic-type X(T), there is a tendency for secondary convergence insufficiency to develop. This is supported by the studies of Hiles^32^ and Chia^33^, who reported an increase in the near deviation of more than 5 PD in 12–34% of patients with long-term observation. In our study, the risk of recurrence was higher when the near deviation was larger than the distance deviation by more than 5 PD in basic-type exotropia. Thus, this may partially explain why RR resulted in less recurrence compared with BLR after long-term observations.

Studies on the relationship between age of onset and surgical response have shown variable and contradictory results^15,34–36^. Several studies have reported that the surgical prognosis of early onset exotropia is poor^34,35^, which is similar to our study, showing a worse surgical outcome in patients with an earlier onset. On the other hand, some authors reported that there was no significant difference in the surgical response between early-onset intermittent exotropia and others^15,36^. Age at the time of surgery has also been studied as a factor that can affect postoperative alignment^15,37–43^. Some reports recommended earlier intervention to get a greater chance of postoperative bifoveal fusion with superior binocular vision and stereoacuity^37–39^. A recent meta-analysis reported that early surgery for X(T) provides a better long-term outcome when patients are younger than 4 years old^43^. However, other studies have advocated a delayed approach, as it may allow more accurate measurements and better results^40,41^. On the other hand, several studies showed no difference in the surgical outcomes in children of different age groups^15,42^.

The relationship between preoperative refractive errors and exotropia have varied among previous studies^41,44,45^. Hyperopia^44^ and myopia^45^ both have been shown to exacerbate X(T). Conversely, refractive error was not associated with surgical results in another study^41^. Generally, hyperopic correction can decrease the demand of accommodative convergence, thus increasing the amount of exodeviation. In addition, children with hyperopia are likely to have poor stereopsis^46^. Similarly, in our study, hyperopia greater than + 2.00 D preoperatively was a risk factor of recurrence in patients with basic-type X(T).

This study is a retrospective study and has several limitations. First, we only included patients with basic-type X(T), and the results of our study cannot be extrapolated to other types of exotropia. Second, there were more patients in the RR group with fixation preference. This was based on our previous report representing better surgical success rates using RR surgery for exotropia in the dominant eye with fixation preference^8^. Third, the surgical dose was not uniform in the BLR group, and 314 of 363 patients had undergone augmented surgery compared with the original surgical table^5,20,47^. However, even though most patients in the BLR group had undergone augmented surgery, there were more recurrences after BLR compared with RR. Our result offers an important insight into the high risk of recurrence after BLR recession, regardless of the dosage, and suggests that MR resection may be more effective in the long-term by generating a passive resistance to stretching while in its resting state.

In conclusion, RR was more successful than BLR and likely to minimize the chances of recurrence at an average of 9.5 years after surgery in patients with basic-type X(T). Patients with a younger age of onset, larger exodeviation at near than at distance, and preoperative hyperopia were related to a higher risk of recurrence after surgery.

## Methods

### Patients

This retrospective study included consecutive patients who underwent surgery for basic-type X(T) by one surgeon (J.-M.H.) between May 2003 and February 2009. Approval for conducting this study was obtained from the Institutional Review Board (IRB) of Seoul National University Bundang Hospital. All aspects of the study protocol complied with the Declarations of Helsinki. All data were completely anonymized prior to access and the IRB ethics committee waived the requirement for informed consent.

We included patients diagnosed with basic-type X(T) before surgery and patients had undergone exotropia surgery. Basic-type X(T) was classified when the measured deviation at distance was within 10 PD of the near deviation. They were divided into two groups according to the type of surgical procedures, the BLR group and RR group. Each patient who was followed for at least 5 years was included.

Patients were excluded if they were under 3 years or over 15 years of age at the time of surgery, or if they had a history of strabismus surgery or other ocular surgery, divergence excess-type exotropia, convergence insufficiency-type exotropia, paralytic or restrictive exotropia, sensory exotropia, coexisting vertical deviation greater than 5 PD, dissociated vertical deviation, simultaneous oblique muscle surgery, simultaneous vertical muscle surgery, adjustable surgery, nystagmus, ophthalmic disease, chromosomal abnormality, or systemic disease that could affect ocular alignment. Patients with A or V patterns, or oblique muscle overactions that did not require surgery were included.

### Preoperative examination

Refractive errors were measured by cycloplegic refraction with cyclopentolate hydrochloride 1% eyedrop. For patients with myopia beyond − 1.00 D, we prescribed spectacles of full cycloplegic refraction. In patients with hyperopia above + 3.00 D, we prescribed spectacles of approximately + 1.00 to + 1.50 D less than the full cycloplegic refraction. Amblyopia was defined as interocular difference of two lines or more by the Snellen visual acuity chart. For refractive amblyopia, appropriate spectacle prescription or occlusion treatment was performed before surgery. The angle of deviation was measured by prism and alternate cover test at 1/3 m and 6 m. The fixation preference and constancy of deviation were determined through repeated examinations of the cover-uncover test. Lateral incomitance was defined as 5 PD or more change in the lateral gaze from the primary position. A pattern exotropia was defined as an increase of at least 10 PD exodeviation during downgaze than in upgaze, and a V pattern as an increase of 15 PD exodeviation or more during upgaze than in downgaze^48^. Stereopsis was evaluated with Randot stereotest and converted into logArcsec equivalents.

### Surgical procedures

Patients were treated under general anesthesia, and all surgeries were performed by the same surgeon (J.-M.H.) based on the largest angle of exodeviation ever measured^17^. Table 3 gives the surgical formula that was used for both groups^47^. From 2005, augmentation surgery was performed in the BLR group with the surgical dosage augmented by 1.0–1.5 mm over the original formula, based on several reports representing better surgical success rates with the augmented BLR surgery in exotropia^5,20^.

**Table 3:** Surgical dosage table for patients with basic-type intermittent exotropia.

Prism diopters	Lateral rectus recession/medial rectus resection (mm)	Bilateral lateral rectus recession (mm)
20	5.0/4.0	5.0
25	6.0/4.5	6.0
30	6.5/5.0	7.0
35	7.0/5.5	7.5

### Postoperative measurements

Postoperative alignment at distance and near in the primary position were measured at 1, 3, 6, and 12 months postoperatively and annually thereafter.

### Main outcome measures

Primary outcomes were determined at the final follow-up examination. A successful outcome was defined as a distant angle of deviation between 5 PD or less esodeviation and 10 PD or less exodeviation. Recurrence was defined as an alignment of > 10 PD exodeviation. Overcorrection was defined as an esodeviation of > 5 PD. Secondary outcome measures were reoperation rates, average number of reoperations, and risk factors associated with recurrence after operation. Factors including gender, type of surgery, age of onset, age at surgery, preoperative hyperopia, preoperative visual acuities, preoperative stereopsis, lateral incomitancy, fixation preference, difference in the angle of exodeviation at near and distance were evaluated^20–22,49^.

### Statistical analysis

Statistical analysis was performed using SPSS version 22.0 software for Windows (SPSS Inc., Chicago, IL, USA). The Student t-test and Pearson chi-square test were used to compare preoperative and postoperative characteristics between groups. Kaplan–Meier survival analysis and the log rank test were used to compare the long-term surgical results. Multivariate logistic regression was performed to identify factors affecting undercorrection at the final visit. P-values of less than 0.05 were considered statistically significant. The data are presented as means ± standard deviation unless otherwise specified.
